# Supplementary material for: Differential Expression of NEK Kinase Family Members in Esophageal Adenocarcinoma and Barrett’s Esophagus
Source: Cancers (Basel). 2023 Sep 30;15(19):4821. doi: 10.3390/cancers15194821 (PMC10571661; doi:10.3390/cancers15194821)
Supplement: Supplementary file 1 [file cancers-15-04821-s001.zip › cancers-2615541-supplementary/Supplementary table and figures.pdf]

Table S1. Primer sequences for NEKs qRT-PCR

Primer Name	Sequence (5'-3')
NEK1 RT-F	TCA CCA CGA AGT TTG GCT TT
NEK1 RT-R	GAA AAC GGG AAG CTA TGC AG
NEK2 RT-F	GGA GGG GAT CTG GCT AGT GT
NEK2 RT-R	CAC CAT CAC TTC GTC TGT GG
NEK3 RT-F	CAG TCC ACT GCC GTC TCA TT
NEK3 RT-R	GGG GGT AAG CAC TTC TGG AC
NEK4 RT-F	TCT TTT GGA GGA GGA GGA TG
NEK4 RT-R	AAC TGG CGA GCT TTC ACA CT
NEK5 RT-F	TCA GCA AAC ATC CTC AGC GT
NEK5 RT-R	CTC TTT CAC CTG GGG CTC TG
NEK6 RT-F	GTG AAG CTC GGT GAC CTT G
NEK6 RT-R	CCG TTC TCA TGG ATC CTC TC
NEK7 RT-F	TCT TAA GCA ACT CAA CCA TCC A
NEK7 RT-R	GGA TAG GTC GCC AGC ATC TG
NEK8 RT-F	ATG GAG AAG TAC GAG CGG AT
NEK8 RT-R	TCA CCA GCT TCT GGT CAG C
NEK9 RT-F	CCA TCC GTT CCA ATA GCA GT
NEK9 RT-R	TGT CCT CTT CTT CAC CAC CG
NEK10 RT-F	TGC GAT GTT AAT TCT GGG GCA
NEK10 RT-R	GAC GTT CAA AAG GCA CCG AA
NEK11 RT-F	CAA GAA AGC CAA ACG AGG AG
NEK11 RT-R	AGA GGA GTT GGG CTT CCA AA

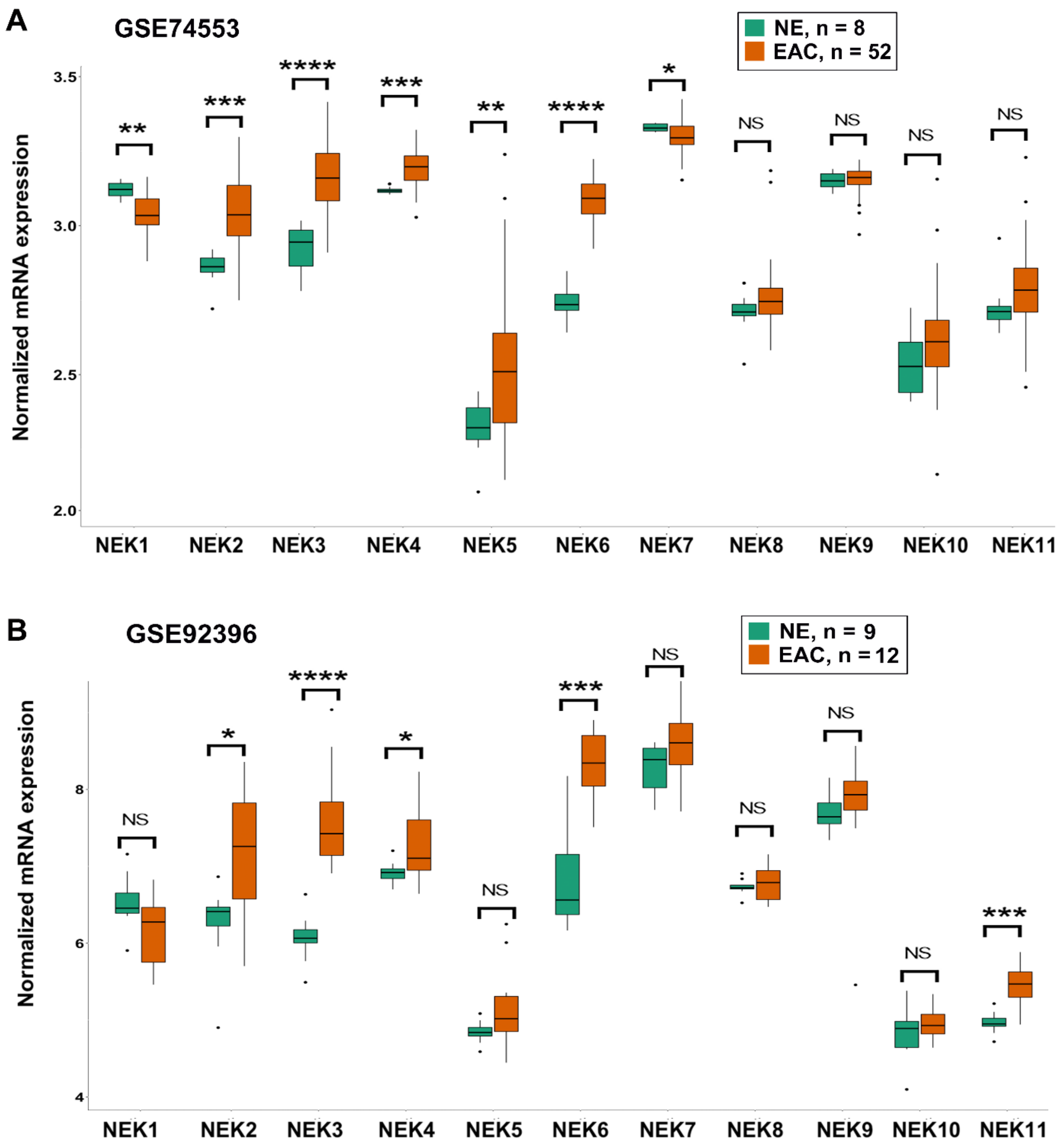

Figure S1. Bioinformatics analyses of NEKs gene expression in normal esophagus (BE) and esophageal adenocarcinoma (EAC)*. GEO datasets GSE74553 (A) and GSE92396 (B) which contain gene expression from normal esophagus and adenocarcinoma samples were analyzed as described in the Methods section. NEKs expressions were presented as normalized values based on original dataset's normalization. *, P<0.05; **, P<0.01; ***, P<0.001, NS, not significance.*

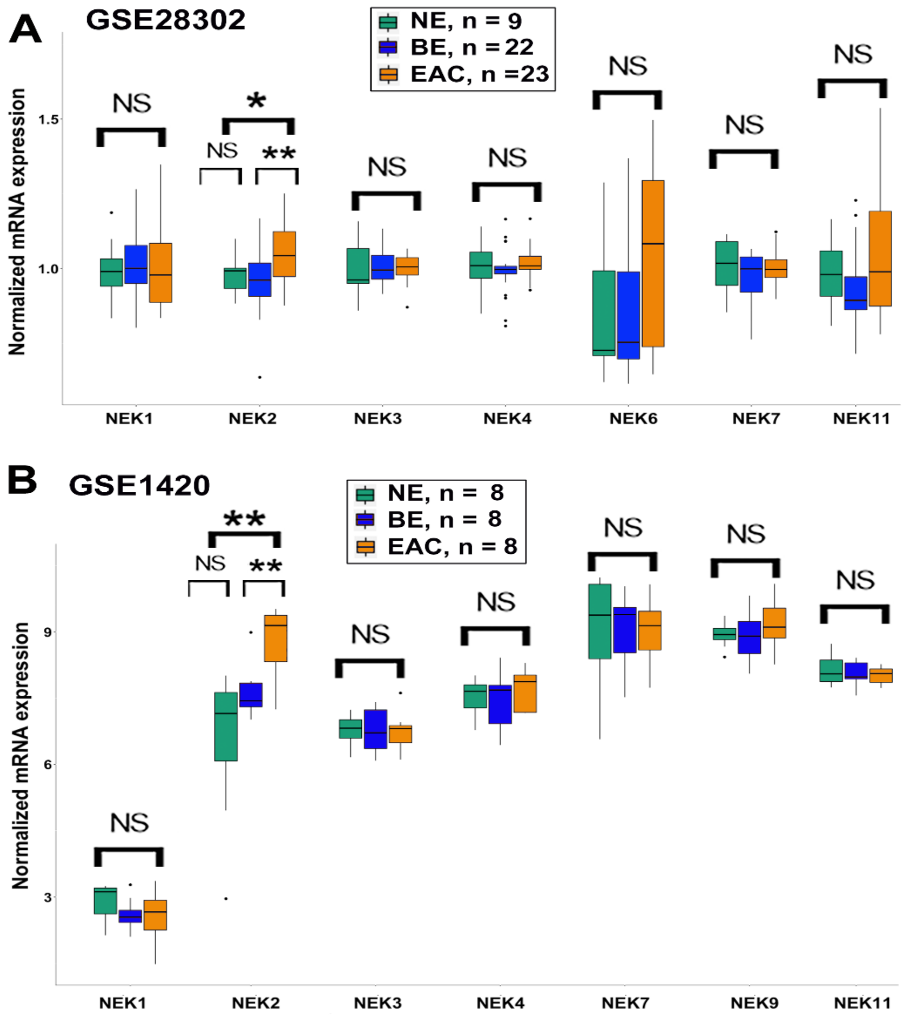

Figure S2. Bioinformatics analyses of NEKs gene expression in normal esophagus (NE), Barrett's esophagus (BE)s and esophageal adenocarcinoma (EAC). *GEO datasets GSE28302 (A) and GSE1420 (B) which contains gene expression from normal esophagus, Barrett's esophagus and adenocarcinoma samples were analyzed as described in the Methods section. NEKs expressions were presented as normalized values based on original dataset's normalization. *, P<0.05; **, P<0.01; NS, not significance.*

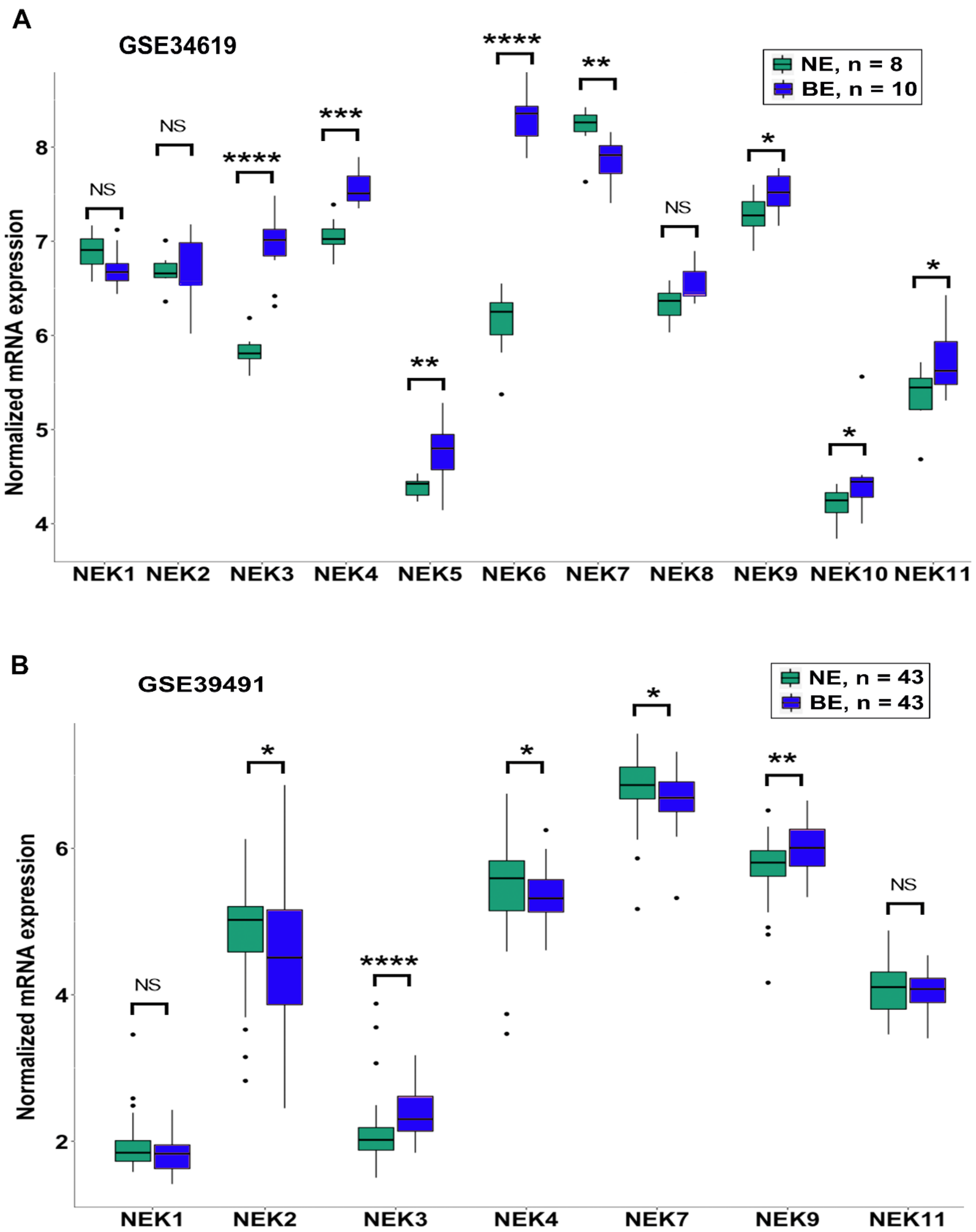

Figure S3. Bioinformatics analyses of NEKs gene expression in normal esophagus (NE) and Barrett's esophagus (BE)*.* *GEO datasets GSE34619 (A) and GSE39491 (B) which contains gene expression from normal esophagus and Barrett's esophagus samples were analyzed as described in Methods section. NEKs expressions were presented as normalized values based on original dataset's normalization. *, P<0.05; **, P<0.01; ***, P<0.001; ****, P<0.0001; NS, not significance.*
