# Supplementary figures and images for: Differential Expression of NEK Kinase Family Members in Esophageal Adenocarcinoma and Barrett’s Esophagus
Source: Cancers (Basel). 2023 Sep 30;15(19):4821. doi: 10.3390/cancers15194821 (PMC10571661; doi:10.3390/cancers15194821)

## For Cancers

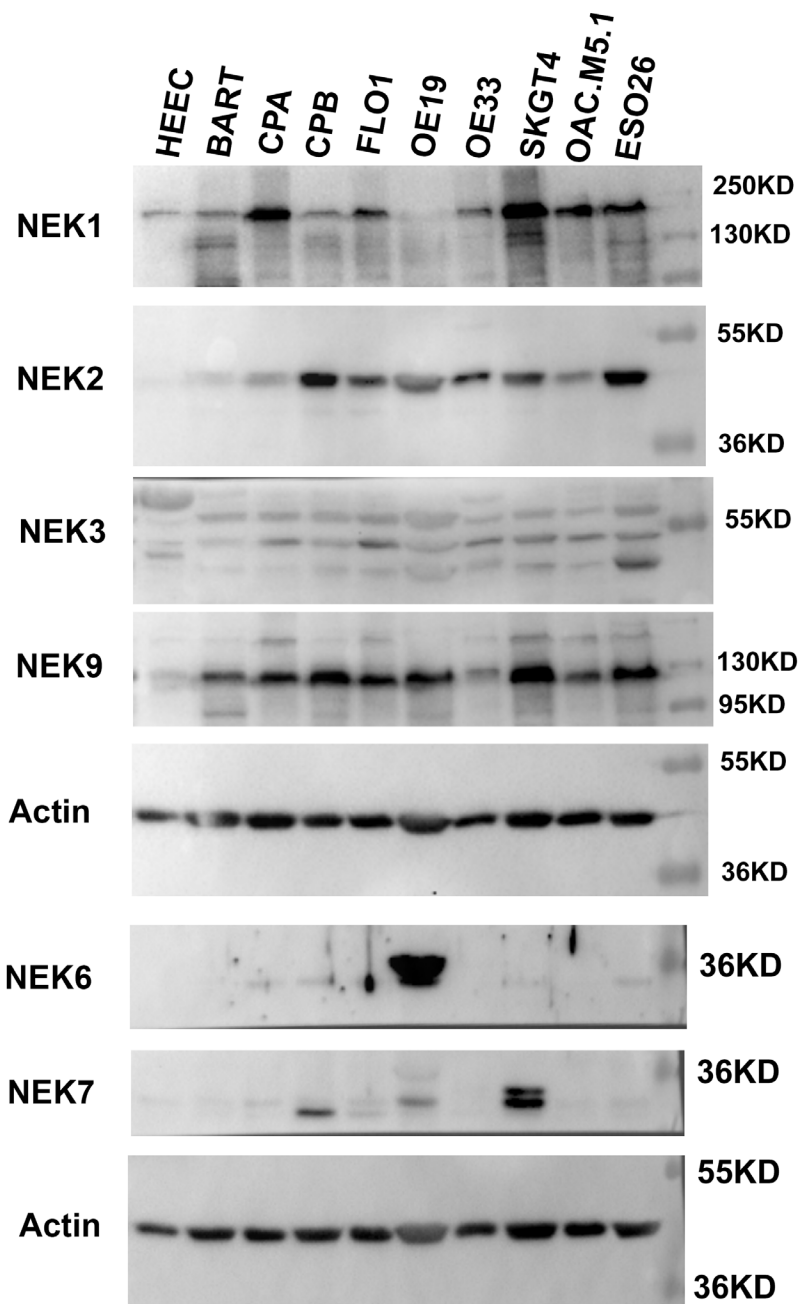

Supplement: Supplementary file 1 [file cancers-15-04821-s001.zip › cancers-2615541-supplementary/File S1_original_images.pdf]
